# Dosimetric characterization of a body‐conforming radiochromic sheet

**DOI:** 10.1002/acm2.12838

**Published:** 2020-02-26

**Authors:** Yi‐Fang Wang, Olga Dona, Kevin Liu, John Adamovics, Cheng‐Shie Wuu

**Affiliations:** ^1^ Department of Radiation Oncology Columbia University Medical Center New York NY USA; ^2^ Department of Chemistry, Biochemistry, and Physics Rider University Skillman NJ USA

**Keywords:** bolus, in‐vivo dosimetry, radiochromic film, radiochromic sheet, surface dosimetry

## Abstract

**Purpose:**

A novel radiochromic PRESAGE sheet (Heuris Inc.) with 3 mm thickness has been developed as a measurement tool for 2D dosimetry. Its inherent ability to conform to irregular surfaces makes this dosimeter advantageous for patient surface dosimetry. This study is a comprehensive investigation into the PRESAGE sheet’s dosimetric characteristic, accuracy and its potential use as a dosimeter for clinical applications.

**Methods:**

The characterization of the dosimeter included evaluation of the temporal stability of the dose linearity, reproducibility, measurement uncertainties, dose rate, energy, temperature and angular dependence, lateral response artifacts, percent depth dose curve, and 2D dose measurement. Dose distribution measurements were acquired for regular square fields on a flat and irregular surface and an irregular modulated field on the smooth surface. All measurements were performed using an Epson 11000XL high‐resolution scanner.

**Results:**

The examined dosimeters exhibit stable linear response, standard error of repeated measurements within 2%, negligible dose rate, energy, and angular dependence. The same linear dose response was measured while the dosimeter was in contact with a heated water surface. Gamma test and histogram analysis of the dose difference between PRESAGE and EBT3 film, PRESAGE and the treatment planning system (TPS) were used to evaluate the measured dose distributions. The PRESAGE sheet dose distributions showed good agreement with EBT3 film and TPS. A discrepancy smaller than the statistical error of the two dosimeters was reported.

**Conclusions:**

This study established a full dosimetric characterization of the PRESAGE sheets with the purpose of laying the foundation for future clinical uses. The results presented here for the comparison of this novel dosimeter with those currently in use reinforce the possibility of using this dosimeter as an alternative for irregular surface dose measurements.

## 1 INTRODUCTION

The dosimetric characteristic of PRESAGE polyurethane dosimeters (Heuris Inc., Skillman, New Jersey, NJ, USA), formulated with a halogenated hydrocarbon free radical initiator and leuco dye has been thoroughly investigated and is well‐characterized.^1^, ^2^, ^3^, ^4^, ^5^, ^6^, ^7^ A 2009 Radiologic Physics Center phantom study investigating the 3D dosimetric applications of PRESAGE showed good agreement (98% pass rate with gamma criteria of 4%, 3 mm) of PRESAGE measurement with EBT radiochromic film (Ashland Inc., Wilmington, DE, USA) measurement for a Head and Neck Intensity Modulated Radiation Therapy (IMRT) plan.^4^ Additionally, PRESAGE was used to measure Gamma Knife Perfexion output factors in 2014.^5^ The excellent agreement between the measurements acquired from PRESAGE and the manufacturer’s Monte‐Carlo based values indicates that PRESAGE is a suitable dosimeter for 3D dosimetry and for clinical research. It is important to mention that small discrepancies were observed between EBT3 film and PRESAGE measurement for low energy applications and small field dosimetry.^7^ The main advantages of using PRESAGE over other types of dosimeters include its linear response to the absorbed dose over a wide dose range, its tissue‐equivalency over a wide energy range, and its capability to provide 3D dose distribution with high resolution. Additionally, the dosimeter can be fabricated to any size or shape for customized clinical and research purposes.

Despite the positive outcomes of using PRESAGE as a 3D dosimeter, 3D dosimetry has not been commonly used in a clinical setting due to the need of specialized equipment to measure 3D dose distributions such as an optical computed tomography^3^, ^4^, ^5^, ^6^ scanner as well as the need of expert users to operate the equipment and analyze the data. Unlike the cylindrical PRESAGE dosimeters, PRESAGE sheets can be scanned with a flatbed scanner similar to the use of EBT3 radiochromic film (Ashland Inc.). The advantage of PRESAGE sheets over EBT3 film is its ability to conform to the patient’s body contours. For instance, the use of radiochromic films for *in‐vivo* dosimetry has been questioned due to the problem of air gaps between the film and surrounding tissue.^9^, ^10^ To avoid the dosimetric errors caused by air gaps, EBT3 films are cut into small pieces and carefully taped when mounted on curved surfaces. Therefore, it cannot be used to measure *in‐vivo* dose distribution over large areas. For dose calibration of the PRESAGE sheets, calibration curves are not necessary for relative dosimetry due to its well‐known characteristic of having a linear response to the absorbed dose. Therefore, unlike radiochromic film, the accuracy of the measurements does not heavily depend on the calibration process. In 2015, a published study by Dumas *et al.* shows the potential use of PRESAGE sheets for dosimetry.^8^ However, in the study, the sensitivity, signal to noise ratio, and temporal stability of the PRESAGE sheets with a different formulation were insufficient for its use in clinical applications. Furthermore, this study did not provide a comprehensive dosimetric characterization of the sheets. For the recently, published reusable PRESAGE 2D sheet,^11^ the Leuco dye cumin‐leucomalachitegreen‐diethylamine (cumin‐LMG‐DEA) was selected due to its rapid decay rate and reusable characteristics while for this study the Leuco Crystal Violet (LCV) was selected to modify the dosimetric characteristic to have greater temporal stability, dose sensitivity and conformality to irregular surface so that the newly developed radiochromic sheets can have stable and accurate dosimetry capability for irregular surface measurement. To distinguish the examined dosimeters from the other PRESAGE dosimeters, the name, PRESAGE‐LCV sheets, is used.

This study investigates the dosimetric accuracy of the newly developed radiochromic sheets concerning their temporal stability of the linear dose response, response to different dose rates, energies, temperatures and angles of incidence, reproducibility, as well as the correction of lateral response artifact (LRA).^13^ In addition to the results from the dosimetric characterization, the measured percent depth dose (PDD) curve was compared with the calculated PDD from the treatment planning system. To investigate the in‐vivo dosimetric capability of measuring dose distributions, regular square field and IMRT field were measured on the surface and compared with the results from EBT3 films. Furthermore, this study provides insights into the dosimetric properties of the PRESAGE‐LCV sheets for its use in future clinical applications.

## 2 MATERIALS AND METHODS

### 2.1 PRESAGE‐LCV sheet formulation/fabrication

**Figure 1:**
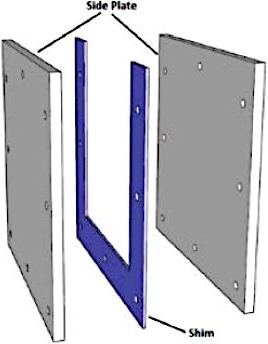
The mold where the formulation of PRESAGE‐LCV sheet was poured

**Figure 2:**
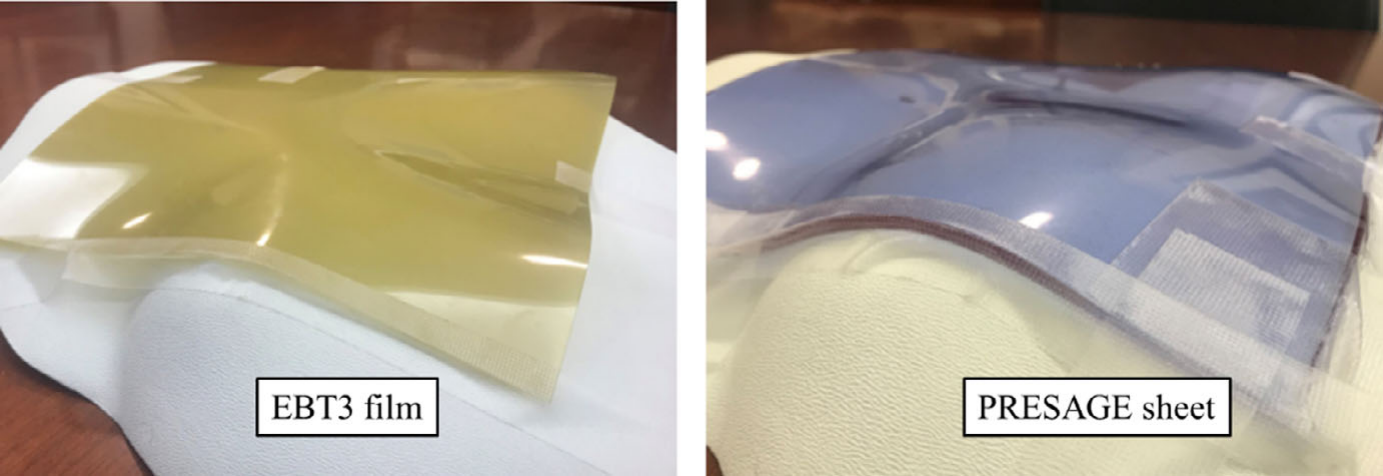
EBT3 radiochromic film and PRESAGE sheet taped on a chest phantom. PRESAGE sheets conform better to irregular surfaces

A mold for the 2D sheets is created by bolting together two polypropylene sheets separated on three sides by a spacer which creates a gap at the top of the mold where the formulation is poured (Fig. 1). A liquid clear aliphatic polyurethane formulation containing LCV, radical initiator is poured into the gap at the top of the mold, then cured at room temperature (RT) under 60 psi pressure for 8–24 h. The sheet is demolded by disassembling the mold. Caliper measurements verified that the sheet thicknesses were ±1.0% of target. The dimension of the sheets investigated is 25 cm by 25 cm by 0.3 cm. To avoid light exposure, the dosimeters were kept in a dark place at RT. In Fig. 2, a single EBT3 film and PRESAGE‐LCV sheet were taped on a chest phantom to demonstrate the differences in conformality.

### 2.2 Dosimetric characterization

A comprehensive investigation into the temporal stability of the linear dose response, energy dependence, dose rate, temperature, angular dependence and reproducibility was performed. The sheets were cut into pieces and irradiated by a linear accelerator, TrueBeam (Varian Medical System Inc., Palo Alto, CA, USA). A flatbed scanner, Epson Expression 11000XL was used to read the transmitted light. Before conducting the experiments, the reproducibility of the response and uniformity of the response at different locations of the scanner was verified. The optical density (OD) was calculated using the red channel of the red green blue scanned image due to its more sensitive response to dose when compared with the green and blue channels.^10^ In addition, 50 dpi was used to accurately measure the dose gradient without sacrificing the signal to noise ratio. To correct the subtle light difference of each scan, an unirradiated sheet was placed beside the measured sheet for each scanning. First, for the evaluation of linear dose response, the optical densities were irradiated at different dose levels (3, 6, 8, 10, 15 Gy) with 4 by 4 cm^2^ field size and scanned at different postirradiation times (0.5, 1, 3, 5, 8, 11, 14, 26, 36, 74 h) and stored at RT. The mean absolute percentage error (MAPE) was employed to assess the accuracy of the linear regression model as a percentage at different scanning times.

$$MAPE=\sum \left|\frac{y-\hat{y}}{\hat{y}}\right|\frac{100\%}{n}$$

where y and $\hat{y}$ are the measured and linear predicted optical density at a specific dose level.n is the number of data points.

For energy dependence and dose rate dependence, 3, 6, 10, 15 Gy was delivered to the dosimeters at different dose rates (100, 600 MU/min) and energies (6 MV, 18 MV, 6 MeV, 12 MeV). Temperature dependence was investigated by placing the dosimeter in contact with a heated water bath (34°C) during the irradiation to simulate *in‐vivo* measurements on the patient’s surface. To acquire optical densities at different dose levels in one piece of the heated PRESAGE‐LCV sheets, virtual wedge with 60° wedge angle was used to deliver a dose pattern with a large dose gradient. The total irradiation time was 20 min. The dosimeter was scanned right after the irradiation. It is noticeable that the simulated temperature and treatment time are much higher and longer than the actual values. The temperature of the patients’ surface is usually under 34°C due to the low temperature of the air in the treatment room and the dosimeter could be placed on top of the clothes instead of contacting with the patient’s skin. Moreover, the overall treatment time is usually under 20 min. For the angular dependence, since PRESAGE‐LCV sheets have uniform composition and properties throughout, the response should not be different with different angles of incidence. To verify the angular independence, the dosimeters were placed in a water tank at the isocenter. The angle between the incident beam and the surface of the dosimeter was varied between 0° and 90° by rotating the dosimeter. Seven sheets were used for seven different angles. 5 Gy was delivered to the isocenter to compare the response. Lastly, to test the reproducibility of the dosimeters, three measurements using different sheets under the same conditions (6 MV, 600 MU/min) were taken. All the analyses were done using MATLAB (MathWorks) and DoseLab (Mobius Inc.).

### 2.3 Correction of lateral response artifact

In this study, the methodology using coefficient correction developed by Lewis *et al.* 13 was applied to correct LRA, a known artifact caused by the scattered transmitted light from the sheets and most importantly, the dependence of reflectivity on the angle of the transmitted light on the mirrors. To measure the correction coefficients, A_L,X_ and B_L,X_ proposed in the article, PRESAGE‐LCV sheets were irradiated at different dose levels (3, 6, 10, 15 Gy) and were scanned at the following lateral positions: −120, −80, −50, 0, 50, 80, 120 mm. For validation of the corrections, 15 by 15 cm^2^ open field was delivered to the dosimeter and the beam profiles were compared. The reproducibility was evaluated by three repeated measurements.

### 2.4 Percent depth dose curve

EBT3 film has a 0.028‐mm thick active layer sandwiched between two 0.125 mm thick layers of polyester while the whole PRESAGE‐LCV sheet is an active layer with a density of 1.07 g/cm^3^. Therefore, the effective point of measurement (the center of the active layer) is at 1.605 mm depth from the surface of the dosimeter. Taking into account the effective depth of measurement, eight pieces of PRESAGE‐LCV sheets were placed at the following depths: 17.1, 27.1, 47.1, 87.1, 137.1, 187.1, 237.1 mm with sheet surface perpendicular to the beam axis. PDD curve was measured using 6 MV photon, 100 cm source to surface distance (SSD) and 4 by 4 cm^2^ field size. The PDD curve was compared with the curve calculated by the treatment planning system (TPS) (Eclipse v15.5, Varian Medical System) used in our clinic.

### 2.5 2D dose distribution measurements

For the verification of 2D dose measurements, measurements in three different scenarios were performed; regular square field measurement on flat surface, on irregular surface and irregular IMRT field on smooth surface without build up. In this study, EBT3 films were employed as the reference dosimeter for 2D dose measurement due to its capability of the stable dose response and fine resolution.^9^, ^10^ However, for the second scenario, irregular surface measurements, EBT3 cannot conform to the surface so TPS calculated dose distribution on the irregular surface was extracted using 3D Slicer. In addition, a 0.5 mm bolus was put on top of the dosimeter for this measurement due to the well‐known inaccurate skin dose calculation of the TPS, Eclipse.^12^

#### 2.5.1 Regular square field measurement on flat surface

A 4 by 4 cm^2^ photon field was delivered to both EBT3 film and PRESAGE‐LCV sheet at 5 Gy. The measured dose difference between the two dosimeters was evaluated using beam profile comparison (field size, penumbra) and 2D gamma analysis with 2%/2 mm criteria.^14^

#### 2.5.2 Regular square field measurement on irregular surface

**Figure 3:**
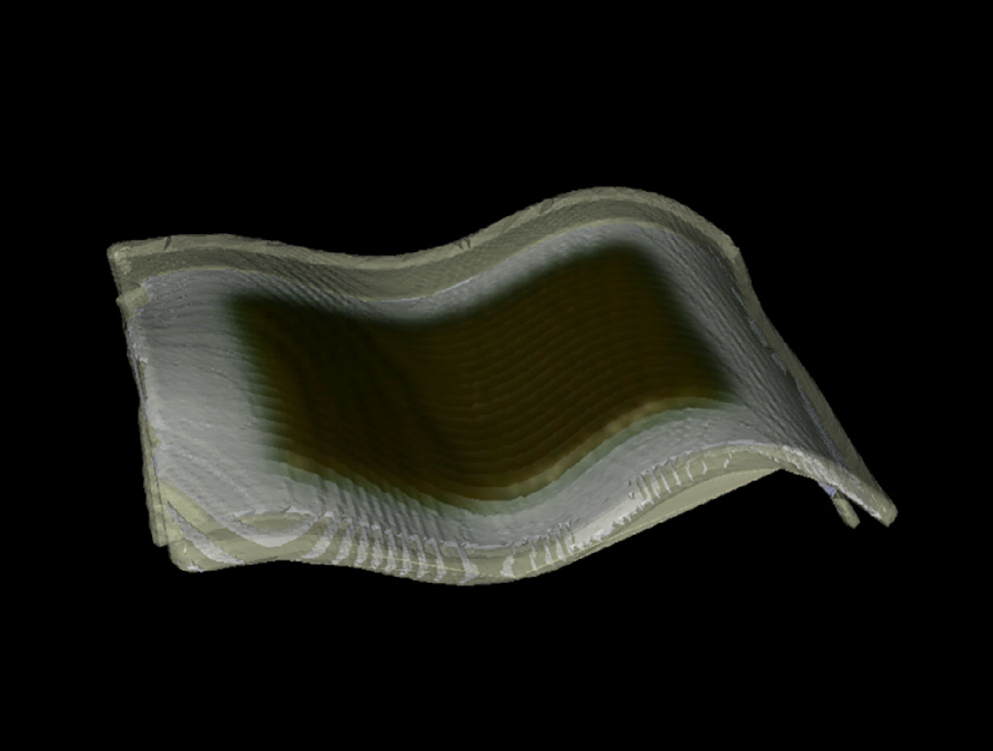
Computed Tomography volume rendering of the measurement setup. A 0.5 mm bolus was put on top of the dosimeter

An 8 by 10 cm^2^ photon field was delivered to a PRESAGE‐LCV sheet conformed to an irregular surface formed by Polypropylene (Fig. 3) with a 0.5‐mm thick bolus on top. The 0.5 mm thick bolus was put on top to avoid the uncertainty in surface dose calculation of the TPS. To compare the calculated dose distribution on the irregular surface from the TPS, the PRESAGE‐LCV sheet was contoured as a structure and the structure was used in 3D Slicer to mask the 3D dose distribution to the 2D dose distribution on the surface of the dosimeter. Finally, the 2D dose distribution in 3D space was mapped to a 2D matrix using MATLAB programming.

#### 2.5.3 Irregular IMRT field on smooth surface

In this section, the capability of the modulated dose measurement of PRESAGE sheets was verified. PRESAGE‐LCV sheets, EBT3 films, and optically stimulated luminescence dosimeters (OSLD) were placed on the surface of an anthropomorphic phantom to measure the dose distribution of a modulated 6 MV photon beam from a Varian Trilogy linear accelerator. NanoDot (Landauer Inc.), an OSL dosimeter, and the microSTARii reader (Landauer Inc., Glenwood, IL, USA) were used in this study. The dosimeters were calibrated using two linear curves in low and high dose regions covering the measured dose range. To verify the accuracy of the calibration curve, two dosimeters were exposed to 3 and 200 cGy. The readings are within 2% of the exposed dose level. In addition, the surface of the phantom is smooth so that a whole EBT3 film can be taped on the surface without air gaps between the film and the surface. Five OSLDs were placed with a 3‐cm spacing separating each OSLD adjacent to the center OSLD vertically and a 4‐cm space separating each adjacent OSLD to the center OSLD horizontally to measure the relative doses at five different points in the field. The point dose measured at the five positions using OSLDs and PRESAGE‐LCV sheets was compared with those measured from the EBT3 films. Moreover, the dose distribution acquired using PRESAGE‐LCV sheet was compared with EBT3 measurement using gamma test with 2%/2mm criteria and histogram of the relative dose difference. Before the comparison, the two dose matrices from the two dosimeters were sampled to the same matrix size, resolution (50 dpi) and rigidly registered. For the histogram analysis, the histogram of the relative dose differences at each pixel was divided by the standard error about the mean of the differences. If the two measured dose matrices agreed within the statistical error of the dosimeters, then the histogram of the dose difference is a normal distribution with a mean at zero and a standard deviation smaller than the statistical error.

## 3 RESULTS

### 3.1 Dosimetric characterization

**Figure 4:**
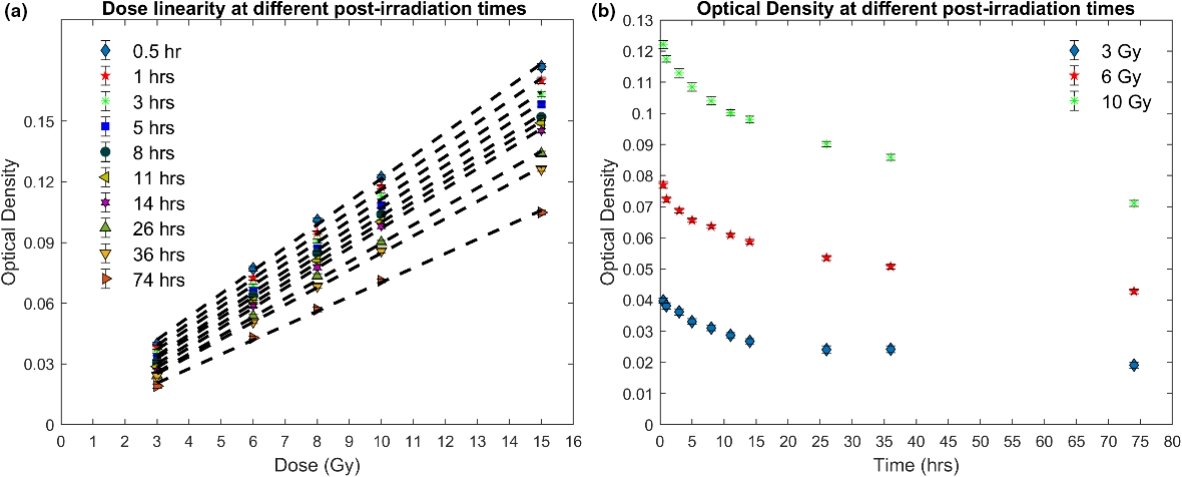
(a) Dose response of radiochromic sheets at different postirradiation times. (b) and dose fading at high and low dose levels

This study examined the dosimetric characteristic and temporal stability of the PRESAGE‐LCV dosimeter. To establish the scanning protocol, net ODs at different dose levels were scanned at postirradiation times ranging from 0.5 to 74 h when stored at RT. The results presented in Fig. 4(a) show the temporal change of the dose linearity. In the examined dose range, PRESAGE‐LCV sheets showed good linear response at all postirradiation times. Positive correlation r = 0.99 (r^2^ = 0.98) was found for the linear responses at all scanning times. MAPE was smaller than 3% if scanned before 5 h after the irradiation. After 5 h, MAPE increases according to time. Table 1 shows the R^2^ values and MAPE of the linear regression at all scanning times. However, the slope of the linear calibration curve decreases as the time increases, which is caused by faster OD fading in the high dose region compared to the low dose region [Fig. 4(b)]. In addition, the decay rate of the optical density is higher at the beginning and then gradually decreases.

**Table 1:** R^2^ values and MAPE (%) of the linear regression curves at different time points

Time (h)	0.5	1	3	5	8
R^2^	0.9976	0.9975	0.9968	0.9968	0.9961
MAPE (%)	2.33	2.58	2.70	2.97	3.79

Time (h)	11	14	26	36	74
R^2^	0.9965	0.996	0.9959	0.9958	0.9937
MAPE (%)	3.65	3.93	4.02	3.70	5.27

Using a 50‐dpi scanning resolution, the standard errors across all values of the measured optical density were minimal. The standard error increases when the delivered dose decreases and the postirradiation time increases due to the lower optical density signals. For doses larger than 6 Gy, standard errors are smaller than ±2% at all scanning times. For 3 Gy, the standard error is ±1.9% if scanned at 0.5 h.

**Figure 5:**
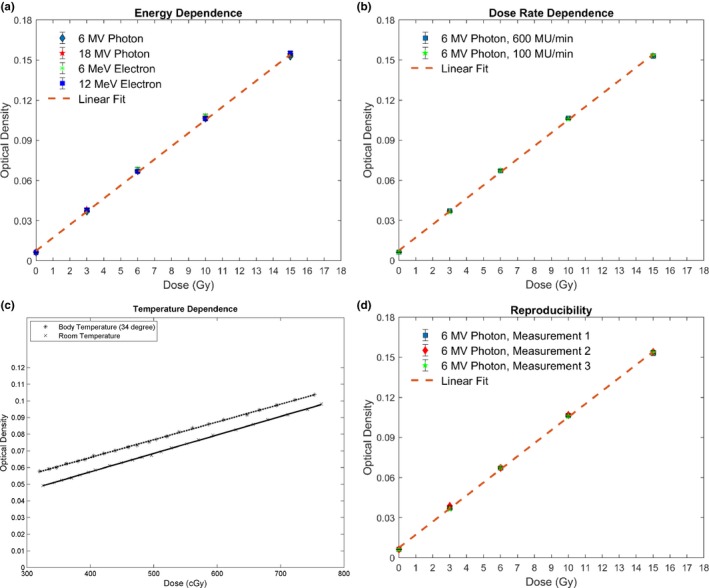
Dose response at different (a) photon and electron energies, (b) dose rates, (c) temperatures and (d) repeated measurements

**Figure 6:**
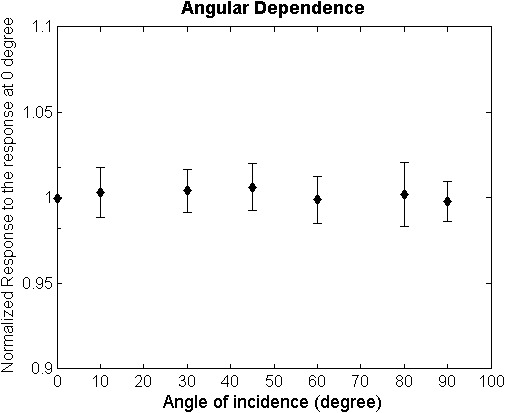
Dose response at different angles between the incident beam and the surface of the dosimeter (0 degree: film surface perpendicular to the beam, 90°: film surface parallel to the beam)

Energy dependence and dose rate dependence were evaluated by plotting the linear curves of different energies and dose rates. The linear curves in Figs. 5(a) and 5(b), ranging from 0 to 15 Gy, show no discrepancy among each other. The maximum deviations of the curves in Fig. 4 were 2.8% at 3 Gy between 6 MV and 12 MeV and 1.8% at 3 Gy between 100 MU/min and 600 MU/min. All the deviations are within the statistical errors of the measurements. For temperature dependence, the same linear dose response was measured while the dosimeter was in contact with a heated water surface during the irradiation [Fig. 5(c)]. The discrepancy between measured and linear modeled OD was smaller than 2% at each point of the curve and R^2^ value, 0.9995. It is noticeable that this study investigated the relative dose distribution measurement rather than absolute dosimetry. Therefore, even though the heated dosimeter measured higher optical density, accurate relative dose measurement can be acquired with the linear dose response. To evaluate the reproducibility of the dosimeter, same measurements were conducted using different sheets from the same batch. In Fig. 5(d), the result shows no discrepancy larger than 2%. In Fig. 6, dose responses of the dosimeter at different angles of incidence were presented. The result shows no statistical difference between different angles. The largest discrepancy 0.6% is smaller than the standard deviation of the measurement, 1.3%.

### 3.2 Correction of lateral response artifact

**Figure 7:**
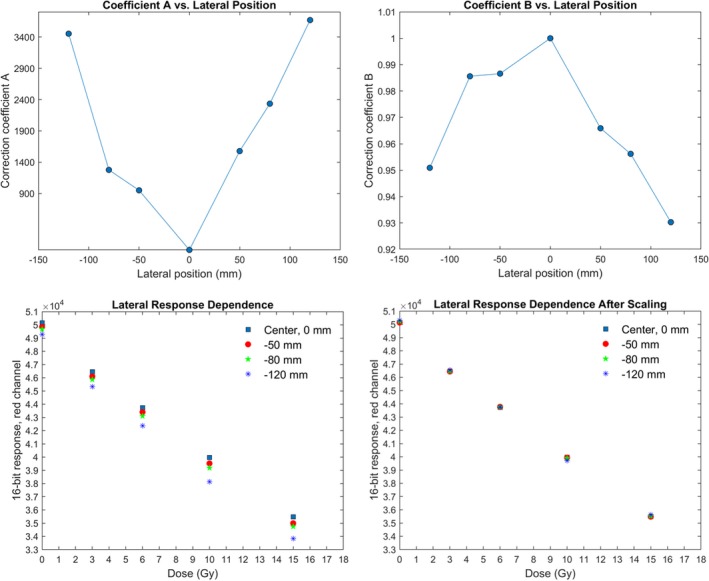
Values of coefficients A and B for the red channel (top right and left) and dose response at different lateral positions before and after scaling (bottom right and left)

**Figure 8:**
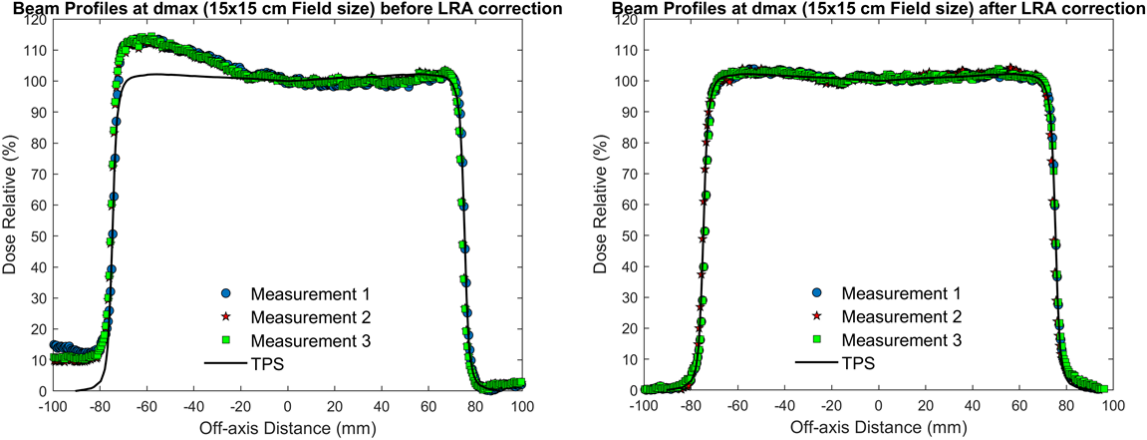
Three repeated beam profile measurements perpendicular to scan direction before and after coefficient correction compared to TPS calculated profiles

In this section, the correction coefficients for the lateral response artifact are reported. It is important to mention that the obtained coefficients (A,B) are scanner‐specific. The behavior of a certain scanner needs to be characterized at each institute. The study by Lewis *et al.* 13 shows that the coefficients are independent of the dose given for a specific lateral position, which corresponds to the results of our study. Figure 7 shows the linear dose response at each lateral position before and after the correction. After the correction, the image intensity difference at all dose levels is within 0.3%. To verify the obtained coefficients, three repeated measurements were conducted using PRESAGE‐LCV sheets irradiated on a 15 cm^2^ × 15 cm^2^ photon field. When scanning the fields, the lateral position of zero was marked on the sheet so that the dose profiles could be plotted according to the lateral position. Figure 8 shows an artifact similar to the one measured by Lewis *et al.* The measured optical density significantly increases toward the lateral edge of the scanner but no difference was observed near the center. After the correction, the maximum relative dose difference between the measurement and TPS was 1.73%.

### 3.3 Percent depth dose curve

**Figure 9:**
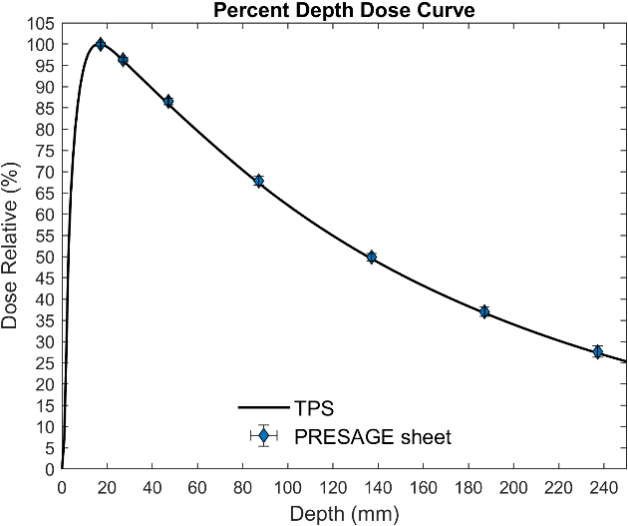
Measured PDD curve from PRESAGE sheet and calculated PDD from TPS

Seven pieces of PRESAGE‐LCV sheets were placed at the effective depth of measurement, 17.1, 27.1, 47.1, 87.1, 137.1, 187.1, 237.1 mm, to measure the PDD curve of a 6 MV photon beam with 4 cm^2^ × 4 cm^2^ field size. The standard errors of Dose Relative (%) (y‐axis) were smaller than ±2% with the largest at the deepest depth, ±1.28%, and the standard errors of Depth (x‐axis) were estimated to be 0.29% including errors from solid water thickness measurements and optical distance indicator. Compared to treatment planning calculated PDD curve, the maximum relative dose deviation was 0.52% at 27.1 mm (Fig. 9).

### 3.4 2D dose distribution measurements

#### 3.4.1 Regular square field measurement on flat surface

**Figure 10:**
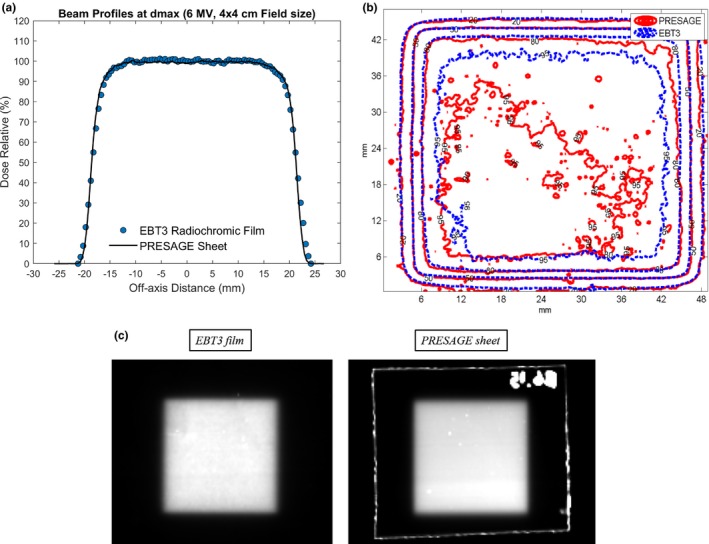
(a) Comparison of beam profiles and (b) 95%, 80%, 50% and 20% isodose lines between EBT3 and PRESAGE sheets for square field, flat surface measurement. (c) Dose images of 4 × 4 square field from EBT3 and PRESAGE

A 6 MV, 4 cm^2^ × 4 cm^2^ regular square field was measured using both PRESAGE‐LCV sheets and EBT3 film. From beam profile comparison, the measured field size and penumbra difference were 0.06 and 0.12 mm, respectively [Fig. 10(a)]. Moreover, the maximum point dose difference was 1.8%. For PRESAGE‐LCV sheets, the normalized optical density image after background subtraction is the relative dose image due to the linear dose calibration curve. The relative dose images of both EBT3 film and PRESAGE‐LCV sheet and isodose line comparison are presented in Figs. 10(b) and 10(c). A passing rate of 99.4% was obtained in gamma test with 2%/2 mm criteria.

#### 3.4.2 Regular square field measurement on irregular surface

**Figure 11:**
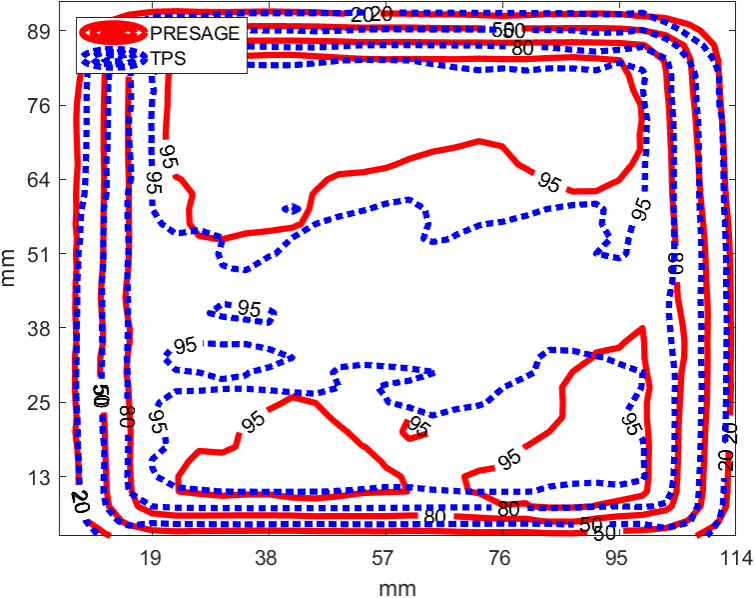
Isodose lines (20%, 50%, 80% and 95%) between EBT3 and PRESAGE sheets for square field, irregular surface measurement

A 6 MV, 8 × 10 cm^2^ regular square field was delivered to PRESAGE‐LCV sheets on an irregular surface with 0.5‐mm thick bolus on top. Figure 11 shows the isodose line comparison of the dose distribution measured and calculated by the TPS. A passing rate of 98% was obtained using 2%/2 mm criteria and the maximum point dose difference was 2.42%. The highest resolution of the TPS is 1 mm so the dose image acquired by the radiochromic sheet was downsampled to 1‐mm resolution for the comparison.

#### 3.4.3 Irregular IMRT field on smooth surface

**Figure 12:**
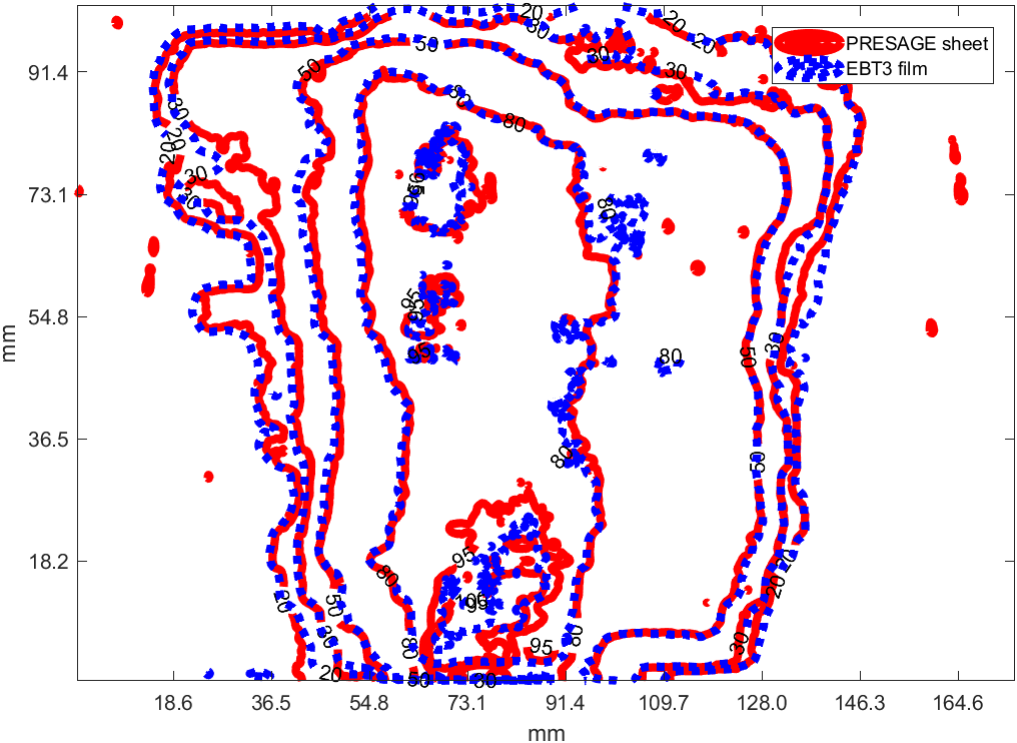
95%, 80%, 50% and 20% isodose lines between EBT3 and PRESAGE sheets for modulated field, smooth surface measurement

**Figure 13:**
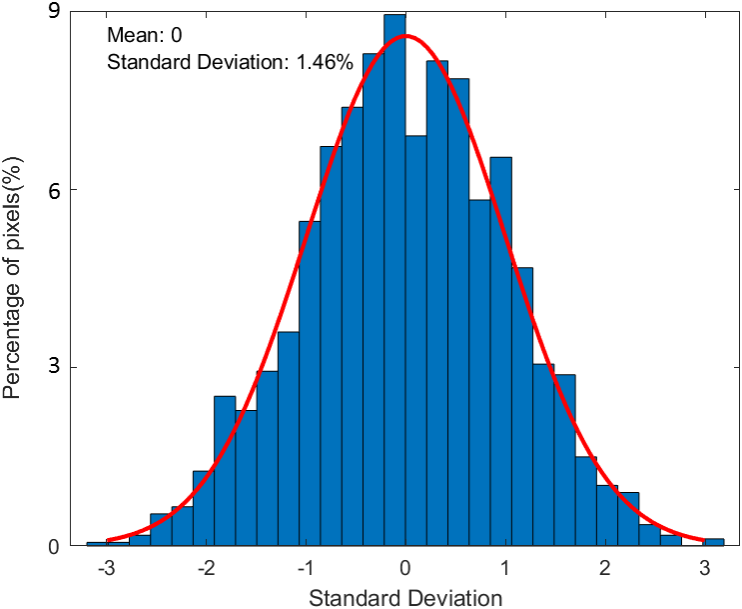
Histogram of relative dose difference between EBT3 and PRESAGE sheets for modulated field measurement

To evaluate the accuracy of relative dose measurement on the surface, the measured dose distribution of a modulated photon field using PRESAGE‐LCV sheets was compared with those from the EBT3 film. Gamma test with 2%/2 mm criteria showed a passing rate of 99.1% (Fig. 12). In addition, the histogram of relative dose difference from pixel subtraction showed a nearly normal distribution with a mean of 0 and a standard deviation of 1.46%, which is within the statistical error of the two dosimeters (Fig. 13).

Additionally, the relative point dose measurements taken at the measured points from both OSLDs and PRESAGE‐LCV sheets were compared to EBT3 films. To obtain the relative dose, the measured doses at five points from the OSLDs were normalized to the OSLD placed in the center. Table 2 depicts the relative dose difference of OSLD and PRESAGE‐LCV when compared to EBT3 film at the five measured points, except for the normalization point. From the results, the OSLDs exhibit a larger discrepancy than PRESAGE‐LCV sheets with their minimum and maximum relative dose difference to be at 3.7% and the relative dose differences exhibited by the PRESAGE‐LCV sheet to be within 1.0%.

**Table 2:** Relative dose difference of OSLDs and PRESAGE sheets at measured points compared with EBT3 film. The doses at five measured points were normalized to the point in the center to obtain relative dose

Relative Dose Difference Compared with EBT3 Film				
Dosimeter	Point 1	Point 2	Point 3	Point 4
OSLD	3.70%	1.80%	2.00%	3.50%
PRESAGE Sheet	0.40%	0.20%	1.03%	0.86%

## 4 DISCUSSION

**Figure 14:**
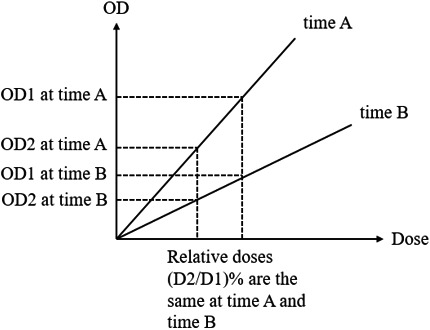
Diagram of the relative dose measurements with linear dose response at different time points

The results from the preceding section show the continuous fading of the measured optical densities of the PRESAGE‐LCV sheets at approximately 2% per hour in the first 5 h, which leads to different slopes of the linear regression curves at different scanning times. In addition, the slope decreases with postirradiation time due to a faster fading in the high dose region compared to the low dose region. However, due to the linear‐response property, the relative dose distribution can be obtained from the distribution of optical density as long as the response curve is linear. The uncertainty of the dose distribution will depend on the degree of fading. Figure 14 shows this important concept that the relative dose measurements are the same using linear response curves with different slopes. For EBT3 radiochromic films and OSLD, signals need to be read out at stable time points when the optical density does not change significantly because the calibration curves of the dosimeters are not linear, which could result in different optical density‐dose relationships if the signals are not stable. In this study, MAPE was used to evaluate the deviation of the measured data points from the linear regression models. It is important to know that MAPE is an estimation of the prediction accuracy of the linear model but not the dosimetric error of the measurement. MAPE increases as the postirradiation time increases due to the fading of optical densities, which is a random chemical process at each pixel. At 5 h after the irradiation, MAPE was measured to be 2.97%. Therefore, it is recommended that the PRESAGE‐LCV sheets are scanned within 5 h after the irradiation. Besides the errors from the modeling, statistical errors also related to the dose delivered to the dosimeter. Lower dose measurements present higher statistical error due to fewer events happening in the dosimeter. The overall statistical errors at most dose levels and scanning times are within ±2%. For 3 Gy, if scanned before 14 h, the standard errors are within ±3%.

The reproducibility of the sheet measurement relates to the uncertainty from both the scanning process and intersheet variability. Unlike for radiochromic film, there is no uncertainty from the PRESAGE sheet calibration curve due to its linear response characteristic. To correct the light intensity difference at each scan, a blank sheet was placed beside each measured sheet and the irradiated sheets were placed at the center of the scanner. In the reproducibility measurement, a maximum of 1.8% difference was observed for three repeated measurements. In addition, no dose rate, angular and energy dependence was observed for PRESAGE‐LCV sheets due to its tissue‐equivalency and uniform composition throughout. The net OD increases as the dosimeters are in contact with a heated surface. However, accurate relative dose measurement can still be obtained due to the remained linear dose response property. For *in‐vivo* measurement on the patient, one can preserve the net OD by not putting the dosimeter directly on the patient’s skin.

A methodology to correct nonuniform response of flatbed scanners for radiochromic film has been previously developed. Two strategies, multi‐channel correction^15^ and coefficient correction^13^ are commonly used to correct the optical density in the radiochromic films. However, multi‐channel correction requires data in the blue channel, which has larger standard errors than the data acquired in the red channel and only mitigates but not entirely removes LRA. This study applied a coefficient correction and showed similar results as what has been shown on radiochromic film corrections.^11^ The effect is evident toward the edge of the measured beam profile, in the direction perpendicular to the scan direction and can be eliminated using the measured coefficient. Please notice that for larger dose distribution measurements, LRA is non‐negligible and should be corrected for accurate dosimetry. The coefficients are unique for each scanner and should be measured at each institute.

Using PRESAGE‐LCV sheets, the measured dose is the accumulated dose measured by the whole active layer. The effective depth of measurement was estimated at the center of the active layer with density correction under the assumption that the dose fall‐off inside the active layer is linear. The results of 6 MV PDD curves show high agreement between PRESAGE‐LCV sheet measurement and treatment planning calculation, indicating the inconsequential influence of volume averaging on dose measurement at the dose gradient.

EBT3 radiochromic film was employed as the reference to evaluate the 2D measurements of the examined dosimeter. Only for the measurement on irregular surface, treatment planning calculated dose was compared as a reference since EBT3 cannot conform to the surface and no dosimeter has been proved to have the capability. For the comparison, if two 2D arrays with statistical errors are similar, the difference between the two should be within statistical errors. Results of the measurements show good correspondence between PRESAGE‐LCV sheet and EBT3, as well as between PRESAGE‐LCV sheet and TPS. For square field measurement on flat and irregular surface, 99.4% and 98% passing rates was obtained using 2%/2mm criteria and the maximum point dose difference in dose profile comparison was 1.8% and 2.42%. For modulated field measurements on the surface, gamma test with 2%/2mm criteria presents a 99.1% passing rate and the histogram of relative dose difference shows a nearly normal distribution with a mean of 0 and a standard deviation of 1.46%. To further map the measured dose distribution to the patients’ skin in the TPS, fiducials can be incorporated into the sheets as landmarks for registration. Programming skills similar to what has been used in Topology could be used to derive a one‐to‐one correspondence between points on curved patient surface and points on the flattened two‐dimensional plane, enabling the PRESAGE sheet to provide skin dose information. It is noticeable that if the shape of the dose distribution changes significantly at different depths, the measurement could be inaccurate due to the thickness of the dosimeter. However, in most of the treatment cases, dose distributions do not change much in 1.6 mm thickness, the effective depth of measurement. Lastly, the examined dosimeter is tissue‐equivalent and has the same bolus effect as a 3‐mm bolus, which could be mitigated by conservative and dosimetric treatment planning techniques.

In addition to the comparison of square fields, modulated dose measurements of PRESAGE‐LCV sheets and OSLDs were compared with EBT3 films. From the results, OSLDs exhibit larger discrepancies than PRESAGE‐LCV sheets due to their comparatively larger statistical and experimental uncertainty, the non‐tissue‐equivalence and changes in the OSLD response as a function of energy and dose.^16^, ^17^ The greater discrepancy in the relative dose measurements between OSLDs could be attributed to the uncertainty in the sensitivity of an individual dosimeter as well as the uncertainty in the performance of the reader used to measure the dose extracted from the OSLDs.^17^ The difference in sensitivity between OSLDs may be ascribed to the slight differences in the amount of dosimetry material (Al_2_O_3_:C) contained in the dosimeter.^17^ In addition, discrepancies may arise from the non‐tissue‐equivalent material (Al_2_O_3_:C). The standard and screened OSLDs were reported to have an overall measurement uncertainty of ±10% and ±5.5%. Furthermore, PRESAGE‐LCV sheets have a resolution comparable to EBT3. Compared to EBT3 films, PRESAGE‐LCV sheets show similar dosimetric capabilities while having increased conformability to irregular surfaces, such as the patient skin. These characteristics make the PRESAGE‐LCV sheets a valuable tool for in‐vivo dosimetry.

## 5 CONCLUSIONS

This study investigates the dosimetric characteristics and the potential use of a novel dosimeter for *in‐vivo* 2D dose measurement. The examined radiochromic sheets can be conformed to the patients’ surface and therefore provide accurate relative dose measurement over large areas. Its dosimetric accuracy is comparable to EBT3 films with negligible energy, dose rate and angular dependence. Compared with OSLDs, the dosimeters exhibit less statistical error and higher resolution. Further investigation into its absolute dosimetry capability and other clinical and research applications, such as a replacement of bolus, will be conducted in the future.

## CONFLICT OF INTEREST

The dosimeters were provided by Heuris Inc. free of charge.
